# Action on neovascular age-related macular degeneration (nAMD): recommendations for management and service provision in the UK hospital eye service

**DOI:** 10.1038/s41433-018-0300-3

**Published:** 2019-03-29

**Authors:** Richard P. Gale, Sajjad Mahmood, Helen Devonport, Praveen J. Patel, Adam H. Ross, Gavin Walters, Louise Downey, Samer El-Sherbiny, Mary Freeman, Simon Berry, Nitin Jain

**Affiliations:** 1 The Action on nAMD Group, Birmingham, UK; 2 The York Hospital, York, UK; 3 Manchester Royal Eye Hospital, Manchester, UK; 4 Bradford Teaching Hospitals NHS Foundation Trust, Bradford, UK; 5 National Institute for Health Research Biomedical Research Centre at Moorfields Eye Hospital NHS Foundation Trust and UCL Institute of Ophthalmology, London, UK; 6 University Hospitals Bristol NHS Foundation Trust, Bristol, UK; 7 Harrogate and District NHS Foundation Trust, Harrogate, UK; 8 Hull and East Yorkshire Hospitals NHS Trust, Hull, UK; 9 South Warwickshire NHS Foundation Trust, Warwickshire, UK; 10 Sheffield Teaching Hospitals NHS Foundation Trust, Sheffield, UK; 11 Simon Berry Optometrist, Durham, UK; 12 Bayer, Reading, UK

## Abstract

This report by a group of UK retina specialists and health professionals considers best practice recommendations for the management of sight-threatening neovascular age-related macular degeneration (nAMD), based on collective experience and expertise in routine clinical practice. The authors provide an update for ophthalmologists, allied healthcare professionals and commissioners on practice principles for optimal patient care and service provision standards. Refinement of care pathways for nAMD has improved access to intravitreal anti-vascular endothelial growth factor therapy but there are still variations in care and reported outcomes between clinic centres. Innovative organisational models of service provision allow providers to better match capacity with increasing demand. The authors review the recent NICE guideline for diagnosis and management of AMD, considerations for switching therapies and stopping treatment and need for regular monitoring of non-affected fellow eyes in patients with unilateral nAMD. Actions for delivery of high-quality care and to improve long-term patient outcomes are discussed. Local pathways need to detail nAMD target time to treat, maintenance of review intervals to ensure proactive treatment regimens are delivered on time and appropriate discharge for patients deemed low risk or no longer benefiting from treatment. Actual visual acuity outcomes achieved and maintenance of the level of vision when disease stability is achieved are considered good measures for judging the quality of care in the treatment of patients with nAMD. Robust community referral pathways must be in place for suspected reactivation of choroidal neovascularisation and rapid referral for second eye involvement. Practical considerations for intravitreal injection therapy are outlined.

## Disclaimer

This publication and the expert roundtable meeting on which the article is based were sponsored by Bayer plc. Prescribing information for Eylea^®^ (aflibercept solution for injection) can be found at the end of the article.

## Introduction

Over the past decade, intravitreal anti-vascular endothelial growth factor (anti-VEGF) therapy has become established as the standard of care for the treatment of neovascular age-related macular degeneration (nAMD), supported by evidence from randomised clinical trials as well as routine clinical practice demonstrating efficacy in preventing visual loss and improving vision [1–4]. Wider access to effective treatment has been accompanied by reported decline in the incidence of blindness and visual impairment from age-related macular degeneration (AMD) [5–8]. A study of treatment outcomes reported annual decreases in the incidence of blindness attributable to nAMD in southeast Scotland following the introduction of intravitreal anti-VEGF therapy, from a peak of 9.1 cases per 100,000 population in 2006 declining to 4.8 cases per 100,000 in 2011, representing a 47% decrease over 5 years [8]. However, AMD remains by far the leading cause of certifications for sight impairment in England and Wales [9].

**Fig. 1:**
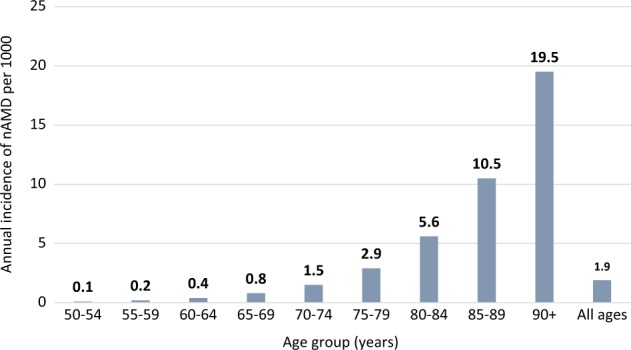
Estimated annual incidence of nAMD per 1000 in the UK population aged ≥50 years. Data from Owen et al. (Web Table 2) [10]. Available at: http://openaccess.sgul.ac.uk/1994/1/bjophthalmol-2011-301109-s3.pdf

**Fig. 2:**
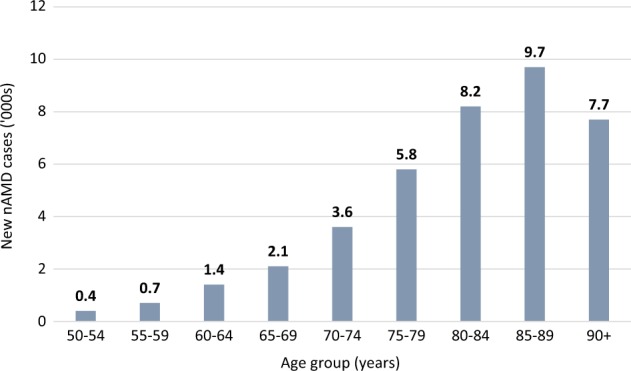
Estimated annual number of new cases of nAMD (‘000s) in the UK population aged ≥50 years. Data from Owen et al. (Web Table 2) [10]. Available at: http://openaccess.sgul.ac.uk/1994/1/bjophthalmol%2D2011%2D301109%2Ds3.pdf

Age-specific prevalence of nAMD in the UK population is estimated at 1.2% in those aged ≥50 years, increasing to 2.5% in those aged ≥65 years and 6.3% in those aged ≥80 years [10]. The number of prevalent cases of late AMD is expected to rise steadily due to population ageing. The annual number of incident cases of nAMD in the UK is estimated at 39,700, a figure that may not reflect the number in need of or likely to benefit from treatment [10]. Figure 1 shows the estimated annual incidence of nAMD per 1000 and Fig. 2 shows the estimated annual number of new cases of nAMD in the UK population aged ≥50 years [10].

Neovascular AMD is a chronic lifelong condition requiring ongoing treatment with regular follow-up and monitoring to control exudative disease activity. An Action on AMD expert group in 2012 previously drew attention to limited or inadequate clinic capacity in the National Health Service (NHS) hospital eye service (HES) that threatened ‘optimal care and access to potentially sight-saving treatment’ for patients with nAMD [11]. As individual nAMD providers differ in structure, size and patient population, as well as in specific service limitations, a flexible approach to service redesign was and remains recommended.

A follow-up Action on nAMD roundtable meeting, involving a group of UK ophthalmic retina specialists and health professionals, was held in March 2018 to review earlier recommendations and progress achieved within the HES, with the aim of providing an informed update on issues of current clinical relevance. The authors review different service delivery options for addressing increasing demand and present practical suggestions for maximising capacity. Recent guideline advice for diagnosis and management of AMD from the National Institute for Health and Care Excellence (NICE) [12] is reviewed and practice considerations for switching therapies, stopping treatment and monitoring of non-affected fellow eyes discussed. The practicalities of intravitreal injection delivery are also revisited. For quick reference, key points and recommendations are summarised in Tables 1 and 2.

**Table 1:** Action on nAMD service provision: key points

*• Recommendations for service providers*
° Maintain high-quality service provision standards without compromise for target time to treat, maintenance of review intervals for timely proactive treatment and appropriate discharge.
° Ensure continuing proactive treatment strategies to maximise and maintain vision benefits.
° Monitor and benchmark treatment outcomes, attendance compliance and discharge rates in the context of service slippage.
° Ensure dedicated ophthalmic IT and failsafe administration support.
• *Practices to meet current and future demand*
° Service providers are encouraged to establish a service model best suited to local circumstances and patient population but which allows patients with nAMD to receive timely and effective treatment with optimal follow-up.
° An efficient MDT with upskilled AHPs helps optimise available consultant resource.
° Examples of good practice and service development include clinical assessments and evaluation of images undertaken by trained AHPs under the supervision of a retinal specialist with expertise in managing nAMD, non-medical healthcare professional-led intravitreal injection services and follow-up clinics in the community for surveillance of treated nAMD patients with quiescent disease.
° Consultation based on SD-OCT images acquired either by community optometrists or AHPs within the HES may help to triage individuals with suspected macular disease and provide faster access to treatment for urgent cases.
° Fast and secure IT links are necessary.
° For nAMD patients with quiescent disease following anti-VEGF treatment, consider the feasibility of utilising community-based optometrists to make decisions about the need for hospital assessment and treatment, subject to ongoing training and consultant-led governance.
• *Group review of NICE guideline for diagnosis and management of AMD*
° NICE technology appraisal recommendations must normally be implemented by the NHS within 90 days of the date of publication of final guidance, unless otherwise specified. By contrast, it is not mandatory to apply the recommendations in NICE guideline NG82.
° NICE does not recommend the routine use of ICGA as part of the diagnostic and therapeutic processes, but acknowledges that it is considered particularly useful for identifying PCV, a subtype of nAMD.
° There is an opportunity to seek commissioning support for antiangiogenic treatment of nAMD patients with starting vision better than 6/12 or if vision is worse than 6/96 in a second eye.
° There may be a role for adjunctive PDT in individual nAMD cases, while laser may be a potential treatment option for extrafoveal CNV lesions.
° Effective low-vision support services are necessary as part of routine care and all medical retina units should have access to LVA services.
° Centres should seek funding for an ECLO service where absent.

*AHPs* allied healthcare professionals, *anti-VEGF* anti-vascular endothelial growth factor, *CNV* choroidal neovascularisation, *ECLO* Eye Clinic Liaison Officer, *LVA* low-vision aid, *HES* hospital eye service, *MDT* multidisciplinary team, *ICGA* indocyanine green angiography, *nAMD* neovascular age-related macular degeneration, *NICE* National Institute for Health and Care Excellence, *PCV* polypoidal choroidal vasculopathy, *PDT* photodynamic therapy, *SD-OCT* spectral domain optical coherence tomography

**Table 2:** Action on nAMD clinical management: key points

• *Switching therapies and stopping treatment*
° Treatment switch to a different anti-VEGF drug may be beneficial in a subset of nAMD patients who have no improvement in vision and no improvement in fluid or pigment epithelial detachment following prior antiangiogenic treatment.
° Decisions to withhold or stop anti-VEGF treatment need to be patient-centred and tailored to the needs of individual patients.
° Discharge from clinic may be considered if there are robust community referral systems in place.
° A structured monitoring programme for specific cohorts of inactive nAMD patients (e.g., better-seeing eyes) meeting local criteria for discharge merits consideration.
• *Monitoring non-affected fellow eyes*
° There is a high burden of second eye involvement in patients receiving treatment for unilateral nAMD and regular monitoring of non-affected fellow eyes is necessary.
° Unilateral nAMD patients extended beyond 8-weekly retreatment might benefit from OCT monitoring at shorter intervals to prevent worse outcomes in the second eye.
° Home monitoring and regular eye tests can help identify subtle changes in visual function that may suggest increasing nAMD activity.
° Fellow eye involvement may be considered when determining an appropriate monitoring interval.
• *Practicalities of intravitreal injection therapy*
° The use of peri-injection antibiotics is no longer recommended; however, practitioners should adhere to local protocol until changed.
° Topical administration of iopidine 1% (in cases known to have IOP spikes post injection) 1 h prior to intravitreal anti-VEGF injection can help reduce the magnitude of a rise in IOP post injection.
° For injection clinics led by AHPs, there should be an appropriately trained clinician available to manage any urgent ophthalmological or medical complication.
° Bilateral intravitreal injections during the same visit must be performed as separate sequential procedures.
° Follow-up injection visits should be coordinated by a failsafe administrator to ensure that all patients receive appointments and retreatments at the appropriate time without undue deferral.

*AHPs* allied healthcare professionals, *anti-VEGF* anti-vascular endothelial growth factor, *IOP* intraocular pressure, *nAMD* neovascular age-related macular degeneration, *OCT* optical coherence tomography

## Action on AMD group: practice trends and reappraisal of service provision

### Practice trends in the clinical management of nAMD

The ophthalmology specialty has experienced considerable growth in outpatient attendances for treatment initiation and ongoing management of nAMD. For example, hospital activity in England for people with a primary diagnosis of AMD was less than 10,000 visits in 2005–2006 compared with over 75,000 visits in 2013–2014 [12].

Medical retina services across the UK NHS have expanded and evolved to meet the large treatment burden and increasing demand for intravitreal injection therapy. Over the past 8 years, there has been progressive investment to support refinement of practice protocols and care pathways to ensure provision and delivery of a high standard of care for people with nAMD and other retinal vascular diseases.

Non-invasive spectral domain optical coherence tomography (SD-OCT) is increasingly adopted as a core tool for diagnosis and follow-up decision making. Another marked trend is the shift toward more proactive treatment protocols using anti-VEGF therapy to improve patient outcomes and better manage the burden of care in terms of injections and visits [13, 14].

In clinical trial settings, pro re nata (PRN) or treatment as needed protocols require regular monitoring and strict retreatment criteria when signs of active neovascularisation are present, e.g., fluid on OCT, new or persistent haemorrhage, decreased visual acuity (VA) attributable to disease activity as compared with the previous examination, or dye leakage or increased lesion size on fundus fluorescein angiography (FFA) [15]. However, actual treatment patterns with PRN regimens in routine practice can follow less stringent retreatment criteria and variable or indeterminate monitoring. As such, a PRN retreatment regimen often tends to lead to undertreatment with an insufficient frequency of anti-VEGF injections, resulting in poorer vision outcomes than those observed in pivotal nAMD randomised controlled trials [13, 16–19].

A treat-and-extend dosing regimen can help reduce treatment burden by extending injection intervals when possible and achieves visual outcomes superior to as-needed treatment regimens [16, 20]. Furthermore, the 1-year results from a large randomised clinical trial suggest that a treat-and-extend regimen is a viable and effective alternative to fixed monthly therapy in treatment-naive nAMD patients [21].

### Recommendations for service providers

Adequate capacity and funding of essential HES macular treatment services remain paramount. Recommendations for high-quality care in nAMD services include the following.

#### Maintain high-quality service provision standards without compromise

Several exemplar models of care delivery have been successfully implemented across many parts of the HES. However, adequate investment remains an issue in maintaining provision of high-quality macular clinic services, particularly with regard to funding for recruitment, equipment and space. Ophthalmology is the second largest provider of outpatient care in the NHS, accounting for 8.1% of all English NHS outpatient attendances in the year to March 2017 (Table 3) [22]. The number of attended outpatient appointments in English NHS hospitals and English NHS-commissioned activity in the independent sector has risen significantly, from 51.9 million in 2006–2007 to 93.9 million in 2016–2017 [22]. It is recommended that there should be no compromise in service provision standards. Key performance indicators and metrics need to be established and agreed locally.

**Table 3:** NHS-funded outpatient activity in England 2016-17: top five treatment specialties with the greatest number of attendances [22]

Treatment specialty	Attendances	Non-attendances (DNAs)	Ratio of non-attendances to attendances
Trauma and orthopaedics	7,779,904	607,881	0.08
Ophthalmology	7,642,363	651,106	0.09
Physiotherapy	5,058,780	490,276	0.10
Diagnostic imaging	4,048,842	49,277	0.01
Obstetrics	3,722,720	277,859	0.07
Top five treatment specialties	28,252,609	2,076,399	0.07
All treatment specialties	93,944,301	7,938,009	0.08

See ref. [22]. © Copyright © 2017, NHS Digital (Health and Social Care Information Centre)

Information from NHS Digital, licenced under the current version of the Open Government Licence. https://digital.nhs.uk/catalogue/PUB30154

Local pathways should detail nAMD target time to treat and maintenance of review intervals to ensure proactive treatment regimens are delivered on time and appropriate discharge for patients deemed low risk or no longer benefiting from treatment. Treatment delay following diagnosis or during ongoing management of active disease may result in clinically significant visual loss [23, 24]. Significant delay throughout the early stages of the nAMD care pathway has been reported [25]. Delays in intended care indicate a lack of capacity within the HES, or more specifically a significant mismatch between ophthalmic capacity and demand [26, 27].

Moorfields Eye Hospital is the largest specialist eye hospital in the UK, which saw 509,052 outpatients in 2014–2015 compared with 396,058 in 2010–2011 [27]. During the same period, outpatient activity in the medical retina subspecialty increased by 36% from 79,226 to 107,888. Davis et al. [27] reported that medical retina, despite being known to have a large follow-up workload, had a smaller percentage of lost to follow-up episodes than the more surgically based subspecialties, suggesting that with appropriate diligence it is possible to manage lost to follow-up events to reduce risk to patients and avoid deterioration of sight. A retrospective study of a consecutive series of patients (*n* = 195) treated with intravitreal injection therapy for nAMD in southeast Scotland between 2013 and 2015 found that the mean time from initial ophthalmic assessment to first intravitreal injection treatment was 31.5 days, considerably longer than the recommended service target of 2 weeks [25]. Moorfields Eye Hospital has developed a telephone text reminder system for all patients 2 days before their scheduled appointment, an approach that may be appropriate especially for elderly people or their relatives [27].

#### Ensure continuing proactive treatment strategies to maximise and maintain vision benefits

The treatment goal is to achieve the best possible VA benefits through early initiation of therapy and maintaining continuous timely retreatment. A proactive treatment approach with a one-stop service where assessment and treatment occur at the same appointment minimises the need for intervening visits, supported by clear pathways that define criteria for disease stability and treatment discontinuation. Furthermore, a one-stop service model reduces the likelihood of disease resurgence that can occur between separate monitoring and treatment appointments. A manageable travel burden for patients and carers may also reduce the potential for missed appointments.

The ideal treatment regimen for anti-VEGF management in nAMD, identified by an international working group of retina specialists, should seek to maximise and maintain VA benefits for all patients, titrate treatment intervals to match patients' needs and treat at each monitoring visit [14].

Treatment extension, when applied according to the licensed treatment posology, allows for injection intervals to be gradually increased in a stepwise manner to maintain stable visual and/or anatomic outcomes. Extension may be considered for eyes without macular fluid on OCT and stable vision [28] or stable chronic fluid at optimal treatment intervals. The criteria for maintaining or shortening treatment intervals are persistent macular fluid with stable vision, recurrent fluid, decrease in vision in the presence of fluid, new macular haemorrhage, new choroidal neovascularisation (CNV) or any other sign(s) of exudative disease activity considered vision threatening in the opinion of the treating clinician [28].

#### Monitor and benchmark treatment outcomes, attendance compliance and discharge rates

Significant variations in injection frequency, follow-up monitoring and reported outcomes have been observed between clinic centres providing the same therapeutic regimen for nAMD [29, 30]. A study by the UK AMD EMR Users Group of 13 UK centres using a set protocol for managing treatment-naive nAMD found that the mean inter-centre VA change from baseline to month 12 varied from +6.9 to −0.6 Early Treatment of Diabetic Retinopathy Study (ETDRS) letters (mean improvement of 2.5 letters) [30]. Differences between centres persisted after adjustment for baseline age, starting vision and treatment and visit frequency. There is also significant variation between centres in the proportion of treated eyes with a VA ≥70 letters at month 12 follow-up.

Clinical audit of outcomes helps benchmark standards of care and impact healthcare quality improvements. The use of multiple outcome measures to evaluate the quality of service provision for nAMD is recommended, covering both VA and process review. Talks et al. [29] recommended the following as a minimum requirement for ongoing service evaluation of treated eyes:

- Proportion of patients with VA <35 and ≥70 letters at baseline and at 1-year follow-up;
- Number of treatments administered in 1 year;
- Rate of retention of patients at 1 year; and
- Discharge policies and futility criteria [29].

A minimum set of outcome measures for tracking, benchmarking and improving care for nAMD has been proposed by another working group of international experts [31]. They recommend standardised measurement of: visual functioning and quality of life (distance VA, mobility and independence, emotional well-being, reading speed and accessing information); number of treatments; complications of treatment; and disease control. VA outcomes of treated eyes should capture data for mean change in VA from baseline and the proportion of eyes gaining, losing and maintaining vision (losing <5 letters). While highly useful as measures of care, the practicalities of delivering these outcome measures in busy NHS clinical practices is open to question.

Service providers nonetheless are encouraged to add or consider additional outcomes to meet their specific requirements. A ceiling effect limits potential for improvement in eyes with good vision at baseline [32]. Actual VA outcomes achieved and maintenance of the level of vision when disease stability is achieved are good measures for judging the quality of care for patients with nAMD treated with anti-VEGF therapy [32]. Each centre should actively monitor and audit service slippage.

Service metrics such as time to first treatment and compliance with intended review intervals may also be used as key performance indicators when benchmarking service standards in a unit.

#### Ensure dedicated ophthalmic IT and failsafe administration support

Electronic Medical Record (EMR) systems are recommended as an integral service component, covering documentation of the management plan and review of results, allowing service providers to monitor the number of new patients assessed each week and annual discharge numbers as well as to track and audit treatment outcomes. Medical retina centres require dedicated ophthalmic information technology (IT) support and failsafe administration systems, preferably with specialised administration personnel, for example, an injection service coordinator. EMR suppliers are encouraged to provide seamless software support.

## Practices for managing capacity issues to meet current and future demand

The UK has fewer consultant ophthalmologists per capita than other major European countries [33]. Driven by an ageing and a growing population, case numbers of nAMD in the UK are predicted to increase by 29% between 2015 and 2025 and by 59% from 2015 to 2035 [34]. Ophthalmologists have introduced a number of successful newer organisational models of ophthalmic care and service redesign to meet current and future case demand (Table 4) [34].

**Table 4:** Practical steps or actions for service improvement in the management of nAMD

Options and practical steps for service improvements in the management of nAMD include, but are not limited to:
▪ Direct electronic referral for suspected nAMD can facilitate rapid access to clinical assessment and imaging and needs to be managed as direct appointment bookings to ensure allocation to the correct clinic.
▪ Trained non-medical AHPs may help triage new patient referrals to fast-track prompt treatment or discharge decision making.
▪ Virtual (without actual consultation) clinic models may be provided in the HES or at peripheral sites, with decisions about treatment made by the consultant at a virtual reporting session or by non-medical AHPs directly. Training, audit and governance must be appropriate.
▪ Decentralised image acquisition for nAMD virtual clinics where community OCT and cameras are already available and IT data transfer systems are available.
▪ Eye departments have successfully implemented AHP-led anti-VEGF injection clinics.
▪ Risk stratification of patients who are no longer receiving active treatment may allow graded discharge options.

*AHPs* allied healthcare professionals, *anti-VEGF* anti-vascular endothelial growth factor, *HES* hospital eye service, *nAMD* neovascular age-related macular degeneration, *OCT* optical coherence tomography

### Anti-VEGF injection clinics led by nurses and other AHPs

Use of non-medical staff for injection administration remains off-label for the licensed intravitreal anti-VEGF drugs. Notwithstanding this, to address significant workload on ophthalmology clinics, allied healthcare professional (AHP)-led intravitreal injection clinics have been established by multiple HES centres. Ophthalmic nurse practitioners, hospital optometrists, technicians and orthoptists play an increasingly significant role in the delivery of routine retina care, with extended roles helping to expand service provision capacity [35–37]. Results show positive patient satisfaction with a nurse-led injection service, with little variation in patient experience when compared to a doctor-led service, and an acceptable safety profile [35, 38].

### Prioritising and simplifying direct referrals for faster access to macular treatment

**Fig. 3:**
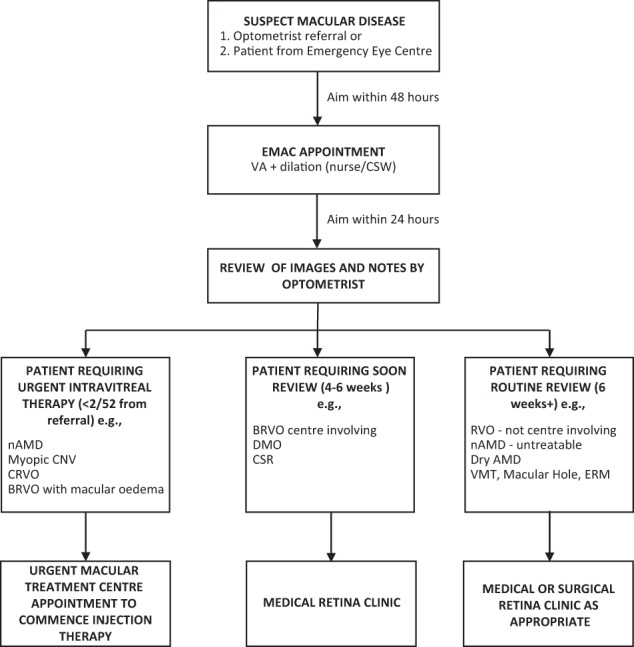
Flow diagram showing Emergency Macular Clinic (*EMAC*) service pathway, Manchester Royal Eye Hospital. *AMD* age-related macular degeneration, *BRVO* branch retinal vein occlusion, *CNV* choroidal neovascularisation, *CRVO* central retinal vein occlusion, *CSR* central serous retinopathy, *CSW* care support worker, *DMO* diabetic macular oedema, *ERM* epiretinal membrane, *OCT* optical coherence tomography, *RVO* retinal vein occlusion, *VMT* vitreomacular traction. Information within flowchart courtesy of Mr Sajjad Mahmood, Manchester Royal Eye Hospital, Manchester, UK

Manchester Royal Eye Hospital has utilised the training of its specialist optometrists and OCT imaging to establish an Emergency Macular Clinic (EMAC), an initiative providing streamlined in-house triage for accurate early diagnosis and prompt access to treatment for individuals with macular disorders. The EMAC offers a rapid access, virtual triage service for people referred directly by high-street optometrists with suspected or confirmed macular problems, without the need for prior OCT assessment or detailed referral forms. It ensures prioritisation and direct referral to the macular treatment centre for urgent treatment of patients with nAMD, central retinal vein occlusion, branch retinal vein occlusion (BRVO) with macular oedema and myopic CNV. Patients with less urgent conditions such as milder cases of BRVO, diabetic macular oedema (DMO) or macular hole are triaged for outpatient review by a medical or surgical retina consultant. The Emergency Macular Clinic service pathway is detailed more fully in Fig. 3. The EMAC functions as a walk-in service, providing same-day assessment if the patient can attend and, as a minimum standard, aims for appointment within 48 h from first contact. A nurse checks VA and an ophthalmic science practitioner carries out an OCT scan. Specialist trained optometrists review OCT scans within 24 h of imaging and decide on the most appropriate onward referral route. This helps to quickly identify cases that require urgent intravitreal treatment to prevent further sight loss. Approximately 80 new referrals are assessed and triaged each month. The main benefit is accurate macular assessment using OCT, allowing faster access to treatment for those with confirmed sight-threatening macular conditions. Key performance indicators are time to first review in EMAC (within 2 days) and time to first treatment (as soon as possible after review in EMAC with a maximum duration of 14 days from the initial referral to EMAC). Manchester Royal Eye Hospital also records and reports other key performance indicators monthly, including compliance with review intervals (within 7 days of intended), complications and patient experience (maximum patient journey including assessment and treatment of 1 h from arrival to departure at the satellite units).

The HES in Sheffield has also been running weekly fast-track clinics for new suspected nAMD referrals, led by highly trained and experienced nurse practitioners. This allows for: discharge at the outset for false positives or further referral to other specialties; referral to Low Vision Aid (LVA) services and the Eye Clinic Liaison Officer (ECLO) for those whose vision falls outside the treatment criteria or for advice and support about potential fellow eye involvement; and counselling, prompt FFA/imaging and information sharing about disease management and treatment for those who are diagnosed with nAMD.

### Virtual (without actual consultation) clinic assessments

Remote evaluation of patients may not only enable earlier detection but also provide for effective monitoring of vision-threatening diseases such as nAMD, diabetic retinopathy and glaucoma [39, 40]. Consultation based on SD-OCT images acquired by community optometrists may help to triage individuals with suspected macular disease and provide faster access to treatment for urgent cases [40]. Assessments may be undertaken by trained optometrists, nurse practitioners or orthoptists who work as part of a team or network under the supervision of a medical retina specialist in the community or hospital. Optometrist-led stable AMD clinics in the hospital setting may also reduce the burden on existing macular treatment services and have been shown to be safe and efficient [41]. Community follow-up by trained optometrists of previously treated and stable nAMD patients (usually patients who have not required any treatment for at least 6 months of hospital follow-up) may also augment hospital macular services, with medical retina consultant-led governance.

Following the introduction of virtual follow-up appointments for monitoring eligible nAMD patients at Leicester Royal Infirmary Retina department, the proportion of patients (*n* = 196) with significant improvement in mean VA (≥15 letters) increased markedly, despite an unchanged PRN treatment regimen (23.1% with an average of 5.9 injections per patient per year for 2012–2013 compared with 6.9% and an average yearly frequency of 5.6 injections for 2010 and 2011) [42]. The mean time between appointments (follow-up or treatment) decreased from a mean 6.9 weeks to a mean of 5.3 weeks. Mean patient waiting time was 71.4 ± 24.1 min for a conventional hospital visit and 47.3 ± 18.6 min for a virtual clinic appointment.

Evidence shows that virtual medical retina clinics can be implemented successfully using existing resources within a HES [43]. Open to non-urgent external referrals and to existing patients in an outpatient clinic, all patients received VA testing, dilated fundus photography and OCT scans, with grading performed by consultants, fellows and AHPs later on. Review of first attendances between September 2016 and May 2017 (*n* = 1729 patients) showed that the main reasons for referral to face-to-face consultation were image quality (34.7%) and detection of potentially treatable disease (20.2%). Of those referred externally, 45.5% were discharged, 37.1% continued in the virtual clinic and 17.4% were brought in for a face-to-face consultation. As observed by Kortuem et al. [43], virtual medical retina clinics offer a promising solution to ensure patients are seen and treated promptly, and may serve as first-line rapid access clinics.

**Fig. 4:**
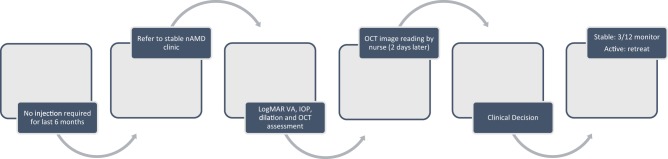
Pathway for nurse-led stable nAMD service reviewing ~40 patients per clinic session, Moorfields Eye Hospital, London, UK. *IOP* intraocular pressure, *logMAR* logarithm of the minimum angle of resolution, *nAMD* neovascular age-related macular degeneration, *OCT* optical coherence tomography, *VA* visual acuity. Information within flowchart courtesy of Mr Praveen Patel, Moorfields Eye Hospital, London, UK

Risk stratification and effective triage are necessary, as is identification of stable patients who may be suitable for monitoring without treatment. A number of centres have implemented stable nAMD clinics (e.g., no treatment given in the previous ~6 to 12 months, depending on local protocol in place) involving nurse-led reading centres for grading of previously acquired OCT scans (Fig. 4).

It is acknowledged that slit lamp examination may reveal additional findings when compared with virtual clinic assessment using combined SD-OCT assessment and colour fundus photography. A service evaluation at Hull and East Yorkshire NHS Trust found that, in a cohort of 242 patients, only 2.5% had isolated retinal haemorrhage undetected with colour imaging and where there were no other OCT features indicating disease activity, arguably an acceptable risk given the benefits of higher throughput with virtual clinics [44].

### Electronic referrals by community optometrists

Timely referral of suspected nAMD can potentially minimise vision loss and maximise possible treatment benefit by providing rapid access to assessment and treatment. The NICE guideline committee commented that there was no evidence to suggest that particular referral pathways are more effective than others, other than the clear benefit of direct referral. However, local areas should have clear pathways in place for referral, discharge and re-referral. In Scotland, an e-referral infrastructure has been established. Electronic referral direct from community optometrists to the HES may help avoid unnecessary outpatient appointments, reduce waiting times and allow patients to be promptly vetted into the correct specialist clinic [45].

### Follow-up and monitoring of quiescent nAMD by community optometrists

Locally commissioned enhanced optometric services provided by accredited community optometrists have proven to be effective for cataract, glaucoma and primary eye care, with patient management commensurate with usual care standards [46]. Collaborative shared care of nAMD offers the potential to reduce workload in hospitals and increase overall capacity to manage the nAMD population more effectively and to manage non-neovascular AMD patients [47]. Devolving responsibilities for monitoring nAMD patients with inactive or quiescent lesions to community optometrists may be a viable option to help release additional capacity within the HES, but this requires ongoing training and quality assurance governance.

Monitoring of quiescent nAMD for reactivation may avoid or prevent irrecoverable sight loss from deterioration of the lesion. Perspectives of shared care for monitoring nAMD indicate that there are concerns about the potential for delays with referrals to secondary care if stable nAMD became active again [48]. Continuous and systematic specialist training for optometrists, under the supervision of ophthalmologists, is required to ensure that optometrists can accurately and confidently elicit the signs of macular disease and thereby minimise unnecessary referrals due to image quality or difficulty with scan interpretation [48].

The Effectiveness of Community versus Hospital Eye Service follow-up for patients with neovascular age-related macular degeneration with quiescent disease (ECHoES) trial concluded that the ability of optometrists, with appropriate training, to make nAMD retreatment decisions from vignettes is not inferior to that of ophthalmologists [47]. Various barriers to implementing shared care pathways for monitoring quiescent nAMD were identified, such as concerns about high-street optometrist competency and potential delays with referrals to secondary care if stable nAMD became active again [48]. The duration of monitoring is not agreed upon but the probability of recurrence declines the longer the treatment-free interval [49].

### Recommendations for maintaining effective local treatment provision

Bespoke approaches to service delivery allow providers to retain flexibility for location and patient population to ensure an efficient nAMD service is made available to patients.

In busy clinical practice, medical retina service models that combine the management of nAMD with other retinal vascular diseases appear to function well. For example, combined intravitreal injection clinics for treating multiple retinal disease entities helps to maintain staff enthusiasm and foster good oversight of indication-specific protocols. Different models of care utilising trained AHPs within the HES can be effective in reducing time from initial presentation to referral, diagnosis and treatment. Separate virtual clinics and assessment is a sound approach to the management of stable nAMD patients and also for those on active treatment.

Fast-track referral models for prompt macular assessment demonstrate that a ‘virtual’ review model relying on interpretation of images by non-medically trained graders is feasible and safe. Further treatment provision is likely to entail intermittent face-to-face clinician consultations in combination with regular clinic assessments and follow-up review of OCT scans and case records. Slit lamp assessment may not be required at every clinic visit for follow-up patients.

Community care models beyond the hospital setting can be effectively established to increase patient throughput capacity but require support and investment in training for primary care eye professionals. Service users need the support of a good transport network. Moreover, adequate space is required and some centres have decommissioned mobile clinic services. A good IT infrastructure is also necessary for this to be effective.

The concept of so-called virtual clinics has been used to develop newer approaches to the management of stable nAMD patients. Colour fundus photographs and OCT images are obtained from patients, with separate remote decision making subsequently by clinicians or AHPs. Virtual reporting sessions allow ophthalmologists and AHPs additional flexibility and time to consult colleagues for challenging or complex cases.

When developing proposed OCT assessment models for nAMD that do not involve face-to-face consultation with ophthalmologists, practitioners should ideally discuss service redesign initiatives with patient representatives and consult service users for non-HES sites.

Colour photography complements virtual OCT imaging sessions. With the growing practice of image assessment and grading by non-ophthalmologists as part of consultant-led multidisciplinary team (MDT) approaches, a service lead should be appointed to oversee continued training, audit and quality assurance strategies to ensure that all staff and team members stay up to date.

Considerations for future service provision include predictive modelling of estimated patient population numbers and expected timings of a likely plateau in workload volume. Embracing newer technology, such as automated OCT analysis or grading and triage by artificial intelligence, may further improve referral quality and allow reliable comprehensive monitoring strategies for long-term patient management.

## Group review of NICE guideline for diagnosis and management of AMD

The latest guideline from NICE aims to improve the speed at which people are diagnosed and treated to prevent sight loss [12]. Table 5 summarises the key NICE recommendations for nAMD and Table 6 outlines the NICE nAMD classification. The terms late AMD (wet active) and late AMD (wet inactive) used in the NICE guideline are referred to in this article as active and inactive nAMD, respectively. The advice from NICE is designed to help optimise service organisation and identification of risk factors, and improve diagnosis, management and review of AMD. However, it is not mandatory to apply the guideline recommendations and the guideline does not override the responsibility of clinicians to make decisions appropriate to the circumstances of each patient [12].

**Table 5:** Brief overview of NICE advice for management of neovascular age-related macular degeneration [12]

*Referral and diagnosis*:
**▪** People with suspected active nAMD must be urgently referred to a macula service, normally within 1 working day.
**▪** People with suspected active nAMD should be offered OCT and FFA to confirm the diagnosis if OCT alone does not exclude neovascular disease.
*Pharmacological management with antiangiogenic therapy:*
**▪** Treatment should be offered within 14 days of referral to the macula service for eyes with a confirmed diagnosis of active nAMD for which antiangiogenic therapy is recommended.
**▪** In eyes with VA of 6/96 or worse (≤25 letters), anti-VEGF treatment for nAMD may be considered only if a visual function benefit is expected (e.g., if the affected eye is the person's better-seeing eye).
**▪** Practitioners are reminded that anti-VEGF treatment for eyes with nAMD and VA better than 6/12 (≥70 letters) is clinically effective and may be cost effective depending on the regimen used, although not currently recommended.
**▪** Practitioners are advised not to offer PDT alone for active nAMD. NICE recommends that PDT is only offered as an adjunct to anti-VEGF therapy as second-line treatment for active nAMD in the context of a randomised controlled trial.
**▪** Intravitreal corticosteroids should not be offered as an adjunct to anti-VEGF therapy for active nAMD.
*Switching and stopping antiangiogenic therapy:*
**▪** Practitioners may consider switching anti-VEGF treatment for people with active nAMD if there are practical reasons for doing so (e.g., if a different medicine can be given in a regimen the person prefers) although the clinical benefits are likely to be limited.
**▪** Observation without giving anti-VEGF treatment is recommended if the disease appears stable.
**▪** Stopping anti-VEGF treatment should be considered if the eye develops severe, progressive loss of VA despite treatment and treatment discontinued if the nAMD is inactive or there is no prospect of functional improvement.
*Non-pharmacological management of AMD: psychological therapies and support strategies:*
**▪** People with AMD and visual impairment are at an increased risk of depression and many may have other significant comorbidities.
**▪** Practitioners are advised to identify and manage depression according to the NICE guideline on depression in adults with a chronic physical health problem.^a^
**▪** Guidance on optimising care for adults with multiple long-term conditions is available in the NICE guideline on multimorbidity.^b^
**▪** Certification of visual impairment should be offered to all people with AMD as soon as they become eligible, even if they are still receiving active treatment, with referral to low-vision services considered for those with AMD causing visual impairment.
*Monitoring of active nAMD, inactive nAMD and those with AMD discharged from hospital services:*
**▪** Active nAMD: offer ongoing monitoring with OCT for both eyes.
**▪** Inactive nAMD: review both eyes at monitoring appointments.
**▪** Discharged:
° Patients should be advised to self-monitor their AMD, consult their primary eye care professional if their vision changes or new symptoms emerge (e.g., blurred or grey patch in their vision, straight lines appearing distorted or objects appearing smaller than normal) and continue to attend routine sight-tests with their community optometrist; and
° A clear local pathway should be agreed covering ongoing management and re-referral when necessary following discharge to primary care.
*Information and support:*
**▪** Information in accessible formats should be provided for people with nAMD, allowing sufficient time to discuss the concerns and questions that the persons have about their diagnosis, treatment and prospects for their vision. Peer support is encouraged.
**▪** Patients should be counselled about the possibility of developing visual hallucinations associated with retinal dysfunction (Charles Bonnet syndrome), a condition that typically improves but can last several years.

*Anti-VEGF* anti-vascular endothelial growth factor, *FFA* fundus fluorescein angiography, *nAMD* neovascular age-related macular degeneration, *OCT* optical coherence tomography, *PDT* photodynamic therapy, *VA* visual acuity

^a^Available at: https://www.nice.org.uk/guidance/cg91

^b^Available at: https://www.nice.org.uk/guidance/ng56

See ref. [12]. NICE © 2018. All rights reserved. Subject to Notice of rights (https://www.nice.org.uk/terms-and-conditions#notice-of-rights)

NICE guidance is prepared for the National Health Service in England. All NICE guidance is subject to regular review and may be updated or withdrawn. NICE accepts no responsibility for the use of its content in this product/publication.

**Table 6:** Classification of active and inactive nAMD, NICE 2018 guideline for diagnosis and management of AMD [12]

nAMD classification	Definition
Late AMD (wet active)	▪ Classic CNV
	▪ Occult (fibrovascular PED and serous PED with neovascularisation)
	▪ Mixed (predominantly or minimally classic CNV with occult CNV)
	▪ RAP
	▪ PCV
Late AMD (wet inactive)	▪ Fibrous scar
	▪ Sub-foveal atrophy or fibrosis secondary to a RPE tear
	▪ Atrophy (absence or thinning of RPE and/or retina)
	▪ Cystic degeneration (persistent intraretinal fluid or tubulations unresponsive to treatment)
	NB Eyes classified as wet inactive may still develop or have a recurrence of wet active AMD

See ref. [12]. NICE © 2018. All rights reserved. Subject to Notice of rights

*AMD* age-related macular degeneration, *CNV* choroidal neovascularisation, *NICE* National Institute for Health and Care Excellence, *PCV* polypoidal choroidal vasculopathy, *PED* pigment epithelial detachment, *RAP* retinal angiomatous proliferation, *RPE* retinal pigment epithelium

In contrast, recommended treatment options outlined in NICE technology appraisal guidance must normally be made available by the NHS within 90 days of the date of publication of final guidance, unless otherwise specified in the guidance. If there is a significant budget impact, the introduction may be phased over a longer period than the standard 90 days. When exercising their judgement, health professionals are expected to take NICE guidance fully into account, alongside the individual needs, preferences and values of their patients. If NICE guidance is considered not applicable to the services provided or commissioned, that decision should be recorded and reviewed should services change or the organisation takes on new services [50].

The need for further diagnostic investigations beyond OCT and clinical assessment, for example, with FFA or indocyanine green angiography (ICGA), will depend on judgement as to whether these will likely alter or delay the management plan. NICE does not recommend the routine use of ICGA as part of the diagnostic and therapeutic processes, but acknowledges that it is considered particularly useful for identifying polypoidal choroidal vasculopathy (PCV), a subtype of nAMD found in approximately 10% of Caucasian nAMD patients [51].

The 2-week target for time from presentation to treatment (total period from referral to diagnosis and diagnosis to treatment) is specified by NICE in the recognition that a shorter delay would maximise the chances of preserving vision, as vision loss from active nAMD can be acute and rapid [12]. This target is consistent with professional guidance from the Royal College of Ophthalmologists [52] and, according to stakeholder feedback from providers and commissioners, should be achievable in all centres. Some centres aim to provide treatment for eligible nAMD cases within 48 h of referral.

Ranibizumab (Lucentis^®^, Novartis Europharm Limited, Dublin, Ireland) and aflibercept (Eylea^®^, Bayer AG, Leverkusen, Germany) are licensed intravitreal anti-VEGF drugs (antiangiogenic agents) indicated for adults for the treatment of nAMD as well as for visual impairment due to other retinal conditions [53, 54]. NICE guidance recommends both agents as first-line treatment options for adults with nAMD who meet the following baseline clinical criteria: best corrected VA is between 6/12 and 6/96, there is no permanent structural damage to the central fovea, the lesion size is less than or equal to 12 disc areas in size and there is evidence of recent presumed disease progression (blood vessel growth, as indicated by FFA, or recent VA changes) [55, 56]. Furthermore, the manufacturer must also provide the drug with the discount agreed in the patient access scheme. Ranibizumab and aflibercept are also accepted for use in adults with nAMD in NHS Scotland [57], NHS Wales and Health and Social Care Board Northern Ireland.

There is an opportunity to seek commissioning support for antiangiogenic treatment of nAMD patients with starting vision better than 6/12 on the basis of current evidence. The guideline from NICE acknowledges that treatment of nAMD patients with initial VA >6/12 may be beneficial and that treatment may be of benefit for those whose vision is 6/96 or worse. Evidence from the UK EMR Users Group supports the immediate treatment of nAMD in patients with initial good baseline vision better than 6/12, representing a cost-effective strategy compared with current guidance of initiating treatment only at a presenting vision of 6/12 or worse [58, 59].

Previous guidance on the use of photodynamic therapy (PDT) for AMD has been updated and replaced by NICE guideline NG82. Practitioners are advised not to offer PDT as an adjunct to intravitreal anti-VEGF therapy as first-line treatment for active nAMD. The authors believe there remains a role in some circumstances for adjunctive PDT in nAMD. Combining PDT with anti-VEGF treatment may provide additional functional benefit over anti-VEGF monotherapy and decrease the burden of intravitreal injections in certain cases of idiopathic PCV [60]. The Action on nAMD group therefore encourages providers to maintain effective dialogue with local commissioners to support clinician judgement for the use of adjunctive PDT for nAMD on an exceptional individual case-by-case basis if there is expected to be a likely benefit. Some practitioners also believe that laser may be a potential treatment option for extrafoveal CNV lesions.

Non-affected contralateral eyes should be scanned whenever the eye undergoing treatment for nAMD is being scanned. Only a limited number of OCT scans may be necessary in the first year of treatment, but careful monitoring for potential fellow eye involvement is advised, for example, by ensuring measures are in place to detect early signs of decreased vision [61]. That said, however, routine OCT imaging of treated eyes may give more information regarding treatment response which could help in determining approach to treatment in year 2 and beyond.

Table 7 shows several known risk factors for AMD [12]. Current smokers should be offered smoking cessation advice and support. Initiation of AREDS vitamin supplements in patients with unilateral nAMD is considered to be cost saving and more effective than no supplement use, according to a study of combined trial and real-world outcomes data by the UK EMR AMD Research Group [62].

**Table 7:** Risk factors for AMD [12]

▪ Older age
▪ Presence of AMD in the other eye
▪ Family history of AMD
▪ Smoking
▪ Hypertension
▪ BMI ≥ 30 kg/m^2^
▪ Diet low in omega 3 and 6, vitamins, carotenoid and minerals
▪ Diet high in fat
▪ Lack of exercise

See ref. [12]. NICE © 2018. All rights reserved. Subject to Notice of rights

*AMD* age-related macular degeneration, *BMI* body mass index

The NICE guideline underscores the importance of information provision as part of a well-organised patient pathway. Low-vision support services assist in the provision of information for those with visual loss, provide practical advice in acute situations and signpost or facilitate access to support services, including social care. The role of the Eye Clinic Liaison Officer is important in this regard, although funding and space in clinics are sometimes barriers to effective provision. Where absent, centres are encouraged to secure funding for an ECLO service to enhance support for people either newly diagnosed with sight loss or patients with an ongoing eye condition. Community LVA provision offers additional potential to ensure people receive the care and support they need, with links to social care.

Furthermore, clinicians need to be mindful of the prevalence of undiagnosed depression and anxiety in nAMD patients and ‘be alert to the opportunity to manage this’ [63]. An observational cross-sectional study by Senra et al. [63] found that 12% of treated nAMD patients showed clinical levels of depression and 17% had clinical levels of anxiety. Depression was more frequent in patients during the early stages of receiving anti-VEGF treatment.

## Switching therapies and stopping treatment

Continued long-term treatment of active nAMD aims to maintain or extend the macular structural and functional gains while attempting to reduce or minimise the treatment burden of clinic visits and injections [28]. Moreover, the success of anti-VEGF treatment depends not just on the treatment of active disease but also on the prevention of disease recurrence and/or worsening [14].

### Defining response to treatment

The need to develop broader consensus on criteria for disease stability, treatment failure and stopping treatment has been recognised previously [64]. Both functional and morphologic responses to anti-VEGF treatment need to be assessed when determining nonresponse or treatment failure. Amoaku et al. [64] suggested that the primary response is best determined at month 4 following treatment initiation with a loading phase of 3 consecutive monthly doses. Secondary response on anti-VEGF maintenance therapy is assessed from month 4 to month 11, with late response determined at month 12 or after. Under the proposed algorithm developed by Amoaku et al. [64], treatment with anti-VEGF therapy should be continued in the absence of morphological or functional deterioration. Poor or nonresponse to anti-VEGF therapy warrants re-evaluation of diagnosis and, if necessary, a switch to a different anti-VEGF agent.

### Switching between anti-VEGF agents for recalcitrant or refractory nAMD

There may be a subset of nAMD patients for whom a switch to a different anti-VEGF agent might be suitable [65], for example, in cases of chronic treatment-resistant exudative disease [66, 67]. Typical clinical reasons for switching agents include persistent or recurrent subretinal and/or intraretinal fluid on SD-OCT and a trend toward decreased vision at 3 to 6 months after starting treatment. Morphological failure characterised by the presence of persistent or recurrent fluid may not necessarily be associated with VA loss.

Outcomes data, largely from uncontrolled retrospective studies, generally indicate significantly improved anatomical outcomes and stabilisation of visual function after treatment switch to another anti-VEGF drug for nAMD patients unresponsive to previous anti-VEGF therapy. However, any short-term functional improvement tends to be limited and transient.

Most of the studies evaluating the potential benefit of changing antiangiogenic therapy in eyes considered insufficiently responsive to previous anti-VEGF treatment report a statistically significant reduction in retinal thickness on OCT, with or without a change in visual function [65]. A database observational study by Barthelmes et al. [68] found no change in mean VA at 12 months of follow-up in eyes that switched from ranibizumab to aflibercept but observed a decrease in the proportion of nAMD lesions that were graded as active. A national EMR database study in the UK assessed visual benefits for nAMD patients who had undergone six continuous monthly ranibizumab injections and were then switched to continuous aflibercept dosing, matched to those on continuous ranibizumab therapy [69]. Following treatment switch, there was a transient significant improvement in VA that peaked at 0.9 ETDRS letters 3 months after the switch, whereas control patients who continued on ranibizumab showed a steady decline in VA.

A meta-analysis of retrospective and prospective switch studies evaluating visual and anatomical outcomes in treatment-resistant nAMD patients switched to aflibercept found a significant improvement in central retinal thickness with vision maintenance or some improvement in VA at 6 months [70].

Factors other than the drug may account for any improvement observed after conversion, such as regression to the mean and change over time [71]. A control group continued on the originally assigned agent would help evaluate how ‘treatment failures’ would do without switching agents.

Duration of the initial loading phase on starting treatment is important, as this may affect results achieved over time [72]. Examination using ICGA is recommended in clear nonresponders where PCV is suspected. Ophthalmologists should retain the option of switching anti-VEGF agents for nonresponders [73]. Switching therapies may also allow clinicians to attempt to increase or extend the treatment interval between injections once response is seen and the disease stabilises. There is no uniform evidence of when to switch; it may be early, usually after several injections or late. It has been suggested that drug tolerance may play a role in a subset of anti-VEGF refractory patients (persistent fluid on OCT despite monthly injections for ≥6 months) who benefit from a treatment switch [74].

### Stopping treatment

Continuation of treatment is recommended if there is persistent evidence of lesion activity, the lesion continues to respond to repeated treatment and there are no contraindications to continuing treatment. Temporary cessation or withholding of treatment may be considered if there is no disease activity (disease has become inactive at ~6 to 12 months), stable disease activity despite frequent and timely dosing and early review (i.e., at 2 weeks to confirm a lack of further response), or there has been one or more adverse events related to drug or injection procedure and the clinician believes that treatment should be withheld. If there is recurrence of CNV, treatment is reinstated until lesion stabilisation is achieved, as indicated by best corrected visual acuity and/or lesion morphology.

Amoaku et al. [64] recommended that ‘treatment should not be discontinued before five consecutive injections have been administered at the optimum recommended dosing interval for the specific anti-VEGF agent, unless there is an obvious deterioration of lesion morphology (poor response) within this period‘. For cases of persistent, unchanged accumulated macular fluid after the loading phase, treatment may be temporarily withheld and reinstated on signs of active neovascularisation with increased fluid on OCT [64].

Response to anti-VEGF treatment for nAMD is heterogeneous. Exclusively denoting treatment success early after several initial intravitreal anti-VEGF injections may overlook a subset of patients who experience a delayed response with continued regular retreatment through 12 months [75]. A retrospective analysis of randomised controlled trial data suggests there may be a subset of so-called delayed responders, defined as those who do not gain ≥15 letters from baseline at month 3 but subsequently gained ≥15 letters from baseline at month 12. These findings reinforce the need to continue treatment in all patients including those with a perceived suboptimal response, provided the patient is gaining or maintaining the visual gains. Definitive treatment success should be determined by the VA outcomes at 12 months rather than 3 months, argued Gale et al. [75].

A consensus approach to treatment decision making in the long-term management of nAMD has been proposed by UK retina specialists [28]. A trial of monitoring without treatment may be considered for eyes with a dry macula and stable vision on 4 consecutive 12-week visits on quarterly anti-VEGF retreatment over 48 weeks. Patients completing a further full year of monitoring without requiring injections and who have no fellow eye involvement over this period may then be considered for discharge from clinic, subject to prompt reinstatement of treatment for disease recurrence or reactivation.

### Recurrence risk during maintenance phase and after stopping treatment

The potential risk of reactivation of nAMD after stopping anti-VEGF treatment warrants close consideration. Studies point to a high rate of reactivation of nAMD over time after disease stability has been achieved [76]. A database observational study of patients receiving anti-VEGF inhibitors using a treat-and-extend protocol by Essex et al. [77] found that the risk of reactivation reached 37.4% for those managed on treatment intervals of ≥20 weeks. The same study reported that 39% of eyes remained inactive at all observed visits during the maintenance phase (defined as starting at the first visit when the practitioner graded the neovascular lesion as inactive) (minimum follow-up of 1 year, mean 945 days) [77].

Continued therapy at capped fixed intervals once disease stability has been achieved may help prevent long-term recurrence in eyes with a good response on anti-VEGF treatment [78].

Patients who have been discharged even after 1 year of disease inactivity would benefit from careful, long-term follow-up. The duration of follow-up may be determined through discussion between eye units and commissioners. The UK AMD EMR Users Group evaluated time to retreatment after a pause in anti-VEGF therapy for intervals of 3 to 12 months in a large dataset of 12,951 eyes from 14 clinic centres [49]. Recurrence risk diminished the longer the treatment-free interval. After a treatment-free interval of 12 months, 34% of these eyes required retreatment after 12 months of follow-up. For all treatment-free intervals, the mean VA at first visit after retreatment was lower than that at the beginning of the treatment-free period, demonstrating that VA did not fully recover on resumption of treatment [49].

Discharge from HES may be considered where local pathways covering re-referral when necessary have been agreed. Fast-track referral pathways should be in place at all times to allow for prompt patient reassessment where there are symptoms suggestive of recurrence or if reactivation is diagnosed by an optometrist or doctor. Evidence is needed on their effectiveness and false negative/false positive referral rates.

Many retina specialists believe that nAMD patients are never truly discharged, with ongoing review required using low-risk clinics or monitoring by community optometrists. Guidance from the College of Optometrists states that, in the absence of clinical indications, optometrists should not examine patients who are being monitored by the HES more frequently than every 2 years [79]. This highlights the need to determine how best to organise, staff and audit community-based review programmes. The HES has a role to play in ensuring the provision of adequate training and support for community optometrists.

Improved surveillance strategies are needed. Some centres, for example, instruct discharged patients to telephone the AMD service directly if they experience deterioration or increased distortion of vision and are then triaged as new patient referrals. A programme to allow for regular monitoring of specific cohorts of patients with inactive nAMD who meet local criteria for potential discharge may be warranted, e.g., involving scheduled OCT monitoring—in the community or HES—at 3-month intervals to screen for reactivation before any significant decrease of vision in better-seeing eyes.

## Monitoring non-affected fellow eyes

The risk of developing late AMD in the second or fellow eye is high, with almost half of eyes at risk requiring bilateral treatment by 3 years [80, 81], and it is therefore crucial to regularly monitor both eyes using OCT to ensure the best visual prognosis. Involvement of the second eye occurred after a median of 282 days, according to findings from a retrospective EMR database study of patients receiving anti-VEGF treatment for nAMD within a large UK tertiary ophthalmic hospital [82].

### In unilateral nAMD patients, the second eye is usually the better-seeing eye

Extended follow-up intervals beyond 8 weeks for unilateral nAMD patients receiving anti-VEGF therapy may adversely affect VA at presentation of nAMD in the second eye, which is usually the better-seeing eye [83] (Table 8). Therefore, patients with unilateral nAMD whose follow-up intervals are extended beyond 8 weeks on a treat-and-extend treatment regimen might benefit from OCT monitoring at shorter intervals to prevent worse outcomes in their second eye or from home monitoring.

**Table 8:** UK AMD EMR Users Group: multicentre results evaluating effect of extended follow-up for unilateral nAMD on VA of second initially unaffected eyes at the time of diagnosis of nAMD in the contralateral eye [83]

OCT review interval for unilateral nAMD	Risk of losing ≥3 lines of VA in second eye (between pre-diagnosis and diagnosis)	
	OR (95% CI)	*P* value
Sudden presentation^a^	–	–
≤4 Weeks	1.32 (0.82, 2.06)	0.24
>4 To ≤8 weeks	1.61 (1.23, 2.10)	<0.001
>8 To ≤12 weeks	2.25 (1.50, 3.32)	<0.001
>12 Weeks	3.47 (2.21, 5.37)	<0.001

Adapted from Burton et al. [83] and reproduced with permission

*CI* confidence interval, *nAMD* neovascular age-related macular degeneration, *OCT* optical coherence tomography, *OR* odds ratio, *VA* visual acuity

^a^Sudden presentation group refers to those patients seen earlier than their anticipated follow-up interval when the second eye was diagnosed, presumed to have presented early due to worsening visual symptoms in the second eye

Prompt intervention following earlier detection of second eye involvement may improve clinical outcomes [84]. Second treated eyes with nAMD often commence treatment with better baseline VA and maintain better VA than first treated eyes [80]. It has been observed that fellow eye involvement may influence the decision on retreatment interval when managing disease reactivation during extension [28].

### Home monitoring and regular eye tests recommended

Currently in most places after discharge from hospital care, nAMD patients self-monitor. Home monitoring, for example using an Amsler chart (grid), can help identify subtle changes in visual function that may suggest increasing nAMD activity. McKibbin et al. [85] suggested that home monitoring tasks—including measurement of near acuity and assessments of environmental distortion and overall visual function—are likely to remain complementary to standard clinical assessment involving ETDRS distance acuity, slit lamp examination and SD-OCT to determine disease activity and retreatment need.

Faes et al. [86] concluded that both the Amsler grid and preferential hyperacuity perimetry showed promising test performance characteristics in ruling out nAMD in the screening setting and could thus help in monitoring disease. The vast majority of patients use a recording chart rather than the conventional Amsler grid that comprises a high contrast white grid on a black background, a fact that may contribute to high false positive rates. Home monitoring needs to be considered in the context of other supporting local arrangements, such as fast-track community referral, walk-in clinics or monitoring by high-street optometrists.

Future study results will provide further evidence regarding the reliability of different tests for monitoring disease progression and for early detection of fellow eye involvement. The Monitoring for neovascular Age-related macular degeneration Reactivation at Home (MONARCH) study will assess whether three different home monitoring vision tests performed by patients with nAMD can detect reactivation of disease with comparable accuracy to routine monitoring of nAMD activity status in HES clinics as part of usual care [87]. Early Detection of Neovascular Age-related macular degeneration (EDNA) is an ongoing UK multicentre study evaluating different test strategies for detecting fellow eye involvement during follow-up in secondary care of people with unilateral nAMD [88].

Similar to diabetic retinopathy screening, there may be a role for screening those either at high risk for first eye involvement or for second eye involvement independently of the follow-up interval when treating unilateral nAMD. A health economic analysis needs to be undertaken to assess the cost effectiveness of such an initiative.

## Practicalities of injection delivery

Anti-VEGF intravitreal injection therapy is usually administered in a dedicated clean room in an outpatient setting with full sterile precautions. It is important that there are standards locally agreed with infection control in place to govern intravitreal injection services in outpatient settings. Guidance from the Royal College of Ophthalmologists recommends that where ventilation to a dedicated clean room has less than 10 air changes per hour, a local risk assessment should be undertaken and agreed with the local infection control team [89]. It is essential that cover is in place to manage any urgent ophthalmological or medical complications. It is advantageous to have nearby facilities for slit lamp biomicroscopy/indirect ophthalmoscopy and viewing retinal images. Resuscitation facilities, based on local risk assessment, should be available in all settings where intravitreal injection therapy is given.

An aseptic technique for intravitreal injection delivery is required to minimise the risk of serious complications. Procedures should ensure adequate anaesthesia and asepsis, including a broad-spectrum microbicide. Sterile gloves should be worn following adequate hand antisepsis and face masks are recommended. Routine use of a surgical drape is no longer considered essential. However, an important principle is that the eyelid margins should always be kept away from the injection site to avoid contamination of the needle [89, 90].

At the pre-injection visit, patients should have a VA measurement (preferably logarithm of the minimum angle of resolution (logMAR)) and clinical evaluation. Immediately prior to the injection procedure, the correct patient identity, correct eye and marking (where dictated by local protocols), evidence of informed consent and the correct drug to be injected must be confirmed. Sedation may be necessary in cases of severe needle phobia or anxiety.

The use of peri-injection antibiotics is no longer recommended, as there is no evidence for prevention of infection and repeated use of topical antibiotics has been shown to increase the occurrence of antibiotic resistance and potentially increased virulence [90, 91]. That said, the local protocol governing administration of intravitreal injection therapy should be adhered to.

It is reasonable to conduct bilateral injections subject to observing appropriate procedures and precautions [52, 92]. When administering bilateral injections during the same visit, each eye must be prepared separately and a different batch of instruments must be used for each procedure. The second injection should be treated as a separate procedure within the same visit. A separate vial from a different batch of the chosen medication is recommended for each eye. Follow-up arrangements for repeat intravitreal injections should be coordinated by a failsafe administrator to ensure that all patients receive appointments and repeat injections at the appropriate time.

To minimise the possibility of reactivation of CNV postoperatively, most practitioners will schedule treatment with intravitreal anti-VEGF injection for 1 to 2 weeks prior to planned intraocular surgery. Recent study findings suggest that cataract operation does not appear to increase the recurrence of CNV in patients with nAMD or to have effect on the time to recurrence and frequency of injections required following surgery [93].

Special precaution is needed in patients with poorly controlled glaucoma and injections should not be given while the intraocular pressure (IOP) is ≥30 mm Hg unless a delay to achieve better pressure control would be sight-threatening. Transient and sustained increases in IOP have occasionally been observed after intravitreal injection procedures. The incidence of sustained elevation of IOP in patients with nAMD reportedly varied from 3.45% to 11.6%, and few patients required surgical management to control IOP [94]. The Diabetic Retinopathy Clinical Research network noted that repeated intravitreal injections of anti-VEGF therapy may increase the risk of sustained IOP elevation or the need for ocular hypotensive treatment in eyes with DMO and no prior open-angle glaucoma [95].

In cases known to have pressure spikes following intravitreal injection, topical administration of iopidine 1% at 1 h prior to anti-VEGF injection can help significantly reduce the magnitude of a post-injection elevation of the IOP [96]. Prophylactic treatment with oral acetazolamide may be considered an option to minimise neuroretinal rim damage in high-risk glaucoma patients who are most vulnerable to IOP spikes and undergoing repeated injections of anti-VEGF inhibitors [97]. Both IOP and the perfusion of the optic nerve head should be monitored and managed appropriately. In the event of a retinal break, intravitreal treatment should be withheld and treatment should not be resumed until the break is adequately repaired.

The benefit–risk profile of anti-VEGF treatment should be discussed with the patient before initiating treatment and each time the treatment regimen is altered. There is a theoretical risk of arterial thromboembolic events (ATEs), including stroke and myocardial infarction, following intravitreal use of VEGF inhibitors [53, 54]. A low incidence rate of ATEs was observed in the ranibizumab and aflibercept clinical trials in patients with nAMD or other retinal vascular diseases, with no major or notable differences observed when compared with the respective comparator or control group [53, 54]. Nonetheless, practitioners suggest it is prudent to exercise caution when treating patients who have had a recent heart attack or stroke and discussion with the patient is advised.

## Closing comments

Today, practitioners have greater experience and evidence supporting what works best with respect to effective care and service provision in the management of nAMD. Ongoing treatment with intravitreal anti-VEGF therapy is required to maintain morphological recovery and long-term stabilisation of visual function in most patients with active nAMD [4]. Hospital eye services have established a hybrid range of initiatives and strategies to streamline service delivery and maximise capacity, i.e., to enable prompt treatment of referrals when indicated and maintenance of personalised follow-up injection intervals to optimise efficacy in the longer term.

Greater participation of non-medical health professionals in the provision of routine retina care, often undertaking tasks previously only performed by specialist doctors, has proven an effective and safe service model with appropriate training and governance that is led by ophthalmologists specialising in retinal diseases. Imaging technologies have improved dramatically, such that SD-OCT is increasingly used for primary diagnosis, evaluation of treatment efficacy and long-term monitoring in the management of nAMD [98]. This has allowed providers to implement multidisciplinary virtual assessment pathways.

Long-term treatment strategies have shifted from predominantly treatment as needed to proactive dosing strategies, allowing greater use of capacity and finite resources. Outcomes still vary between clinic centres and therefore consistent standardised audit is recommended as a core service standard, preferably using an integrated EMR system. The issue of securing adequate funding remains paramount. Locally agreed metrics need to be agreed to identify why or where additional potential funding may be required. Greater consensus amongst ophthalmic retina specialists is developing with regard to the core outcome standards expected of a high-quality nAMD service.

In conclusion, the group’s suggested actions for delivering high-quality care in the management of nAMD are focussed on three main areas: maintain continuous proactive treatment protocols and strategies to manage the increasing burden of care while minimising monitoring visits; strengthen and extend the skills and roles of non-medical AHPs to maximise HES capacity and multidisciplinary competency to meet a continuing increase in demand (face-to-face patient–consultant consultations should take place at least every 6 to 12 months); and develop a long-term management protocol for anti-VEGF maintenance therapy of nAMD, incorporating expert guidance for monitoring without treatment, stopping treatment and discharge decision making.

Medical retina services will continue to adapt and evolve over time, based on sharing of best practice and continued audit of clinical outcomes and service delivery performance.
